# Prevalence and Incidence of Memory Complaints in Employed Compared to Non-Employed Aged 55–64 Years and the Role of Employment Characteristics

**DOI:** 10.1371/journal.pone.0119192

**Published:** 2015-03-05

**Authors:** Kelly J. Rijs, Tessa N. Van den Kommer, Hannie C. Comijs, Dorly J. H. Deeg

**Affiliations:** 1 Department of Epidemiology & Biostatistics, EMGO Institute for Health and Care Research, VU University Medical Center, Amsterdam, The Netherlands; 2 Department of Psychiatry, EMGO Institute for Health and Care Research, VU University Medical Center, Amsterdam, The Netherlands; 3 GGZ InGeest partner of VU University Medical Center, Amsterdam, The Netherlands

## Abstract

**Objectives:**

To examine the association of employment status and characteristics with prevalent and incident memory complaints (MC) in 55–64-year-olds.

**Methods:**

Subjects were participants of the Longitudinal Aging Study Amsterdam (LASA). Respondents with baseline data were selected to examine the association of employment status (n = 1525) and employment characteristics (n = 1071) with prevalent MC (i.e., MC at baseline). Respondents without MC at baseline were selected to examine the association of employment (n = 526) and employment characteristics (n = 379; working hours, job prestige, job level, psychological job demands, iso-strain) with incident MC (i.e., no MC at baseline and MC at three-year follow-up). Associations were adjusted for relevant covariates (demographics, memory performance, physical health, mental health, personality traits). Logistic regression was applied. Data were weighed according to gender and age of the Dutch population.

**Results:**

At baseline 20.5% reported MC. At three-year follow-up, 15.4% had incident MC. No associations were found between employment status and MC. Adjusted analysis revealed that individuals with high occupational cognitive demands were more likely to have prevalent MC.

**Conclusions:**

Middle-aged workers are equally as likely to experience MC as non-working age-peers. Among workers, those with cognitively demanding work were more likely to experience MC, independent of memory performance. Memory decline due to ageing may be noticed sooner in 55–64-year-olds performing cognitively demanding work.

## Introduction

Memory complaints may lead to restricted daily functioning [1] and decreased quality of life [2]. Memory complaints have been shown to be associated with poor memory performance [3, 4] and memory decline [3, 5]. However, memory complaints are well-known to not always reflect poor, objectively measured memory performance (e.g. [6]). A wide range of factors, including physical and mental health problems [4, 6], may underlie or cause memory complaints in middle-aged and older individuals. Knowledge of these factors is of importance before an intervention is put into place. Possible underlying or causal factors especially relevant for individuals on the threshold of old age and at the same time still eligible for the work force (i.e. 55–64 years) are employment status and employment characteristics. However, these factors have scarcely been examined. In recent years, psychosocially and cognitively demanding jobs have become more prevalent and physically demanding jobs less prevalent [7, 8]. Work has therefore more often become demanding for cognitive performance, as opposed to for instance physical performance, and possibly result in memory complaints. This fact, combined with a potentially declining memory performance of middle-aged individuals, underlines the need for research on whether employment and employment characteristics are indeed an underlying factor of memory complaints.

Previous research provides indirect clues that memory complaints in 55–64-year-olds may be related to employment status. Mol and colleagues [2] show that memory complaints more often lead to lower satisfaction with life in people aged 55 to 69 years, including employed individuals, compared to 69 to 91 year-olds. Aarts and colleagues [9] report that 55–69-year-olds with multimorbidity, also including employed individuals, are more likely to experience memory complaints compared to older individuals with multimorbidity. One explanation may be that failing memory is expected and acceptable for older but not for younger individuals, more often leading to complaints about memory [9] and a lower satisfaction with life in younger individuals [2]. Another explanation may be that these 55–69-year-olds are still employed for which they may require good memory capacities. They may, therefore, more likely be confronted with cognitive ageing, resulting in more memory complaints [9] and lower life satisfaction from forgetfulness [2]. To our knowledge, only one study examined the direct association between employment status and memory complaints. No association was found in individuals aged 20 to 85 years [10]. These authors, however, examined subjects who attended a Memory Clinic. It may be argued that memory complaints of these individuals are more severe and they may therefore not represent the general population, emphasising the need for additional research.

Amongst individuals aged 59–64 years with memory complaints, stress, and multitasking were most often mentioned as the cause of their memory complaints [11]. Some interviewees specifically mentioned that it was their job that caused the experienced stress and the need for multitasking. Potter, Hartman, and Ward [12] found that high perceived stress, not specifically work stress, was associated with having memory complaints in women aged 60 and over. These studies suggest that perceived stress and multitasking from work may result in memory complaints. Stenfors and colleagues [13] revealed that psychological job demands (e.g. having to work fast or not having enough time) were associated with more cognitive stress symptoms, which include memory complaints, while psychosocial job resources (e.g. work autonomy) were associated with less cognitive stress symptoms. They, however, did not adjust for objectively measured cognitive performance.

In the present study, the goal is to examine whether employment status and characteristics are associated with memory complaints in Dutch middle-aged individuals (55–64-year-olds). In order to accomplish this goal, we first examine whether employment status is associated with prevalent memory complaints and predict incident memory complaints after three years. Second, for individuals who are employed at baseline, we examine whether employment characteristics are associated with prevalent and incident memory complaints. We aimed to examine whether employment (characteristics) are independent underlying factors of memory complaints, so adjustments were made for memory performance as well as other underlying factors (e.g. demographics and physical disease).

## Materials and Methods

### Study sample

Data from the Longitudinal Aging Study Amsterdam (LASA), a longitudinal cohort study, were used. A sample of men and women, aged 55–85 years, stratified by age and sex according to expected 5-year mortality, was drawn from population registries in eleven municipalities in three geographical regions of the Netherlands. Respondents had given their informed consent and underwent face-to-face interviews at their home. In total, 3,107 predominantly Caucasian (>99%) respondents were enrolled in the baseline examination in 1992–93. A second birth cohort of 1,002 respondents was added in 2002 (aged 55–64) from the same sampling frame as the original birth cohort from 1992. Follow-up measurements in both cohorts took place every three years. The study was approved by the Medical Ethics Committee of the VU University Medical Center. Respondents were asked to fill in informed consent forms, in which they state that they have been adequately informed about what it means to participate in the LASA study and that they agree to participate. The sampling, data collection procedures, and non-response have been further described in detail elsewhere [14].

**Fig 1:**
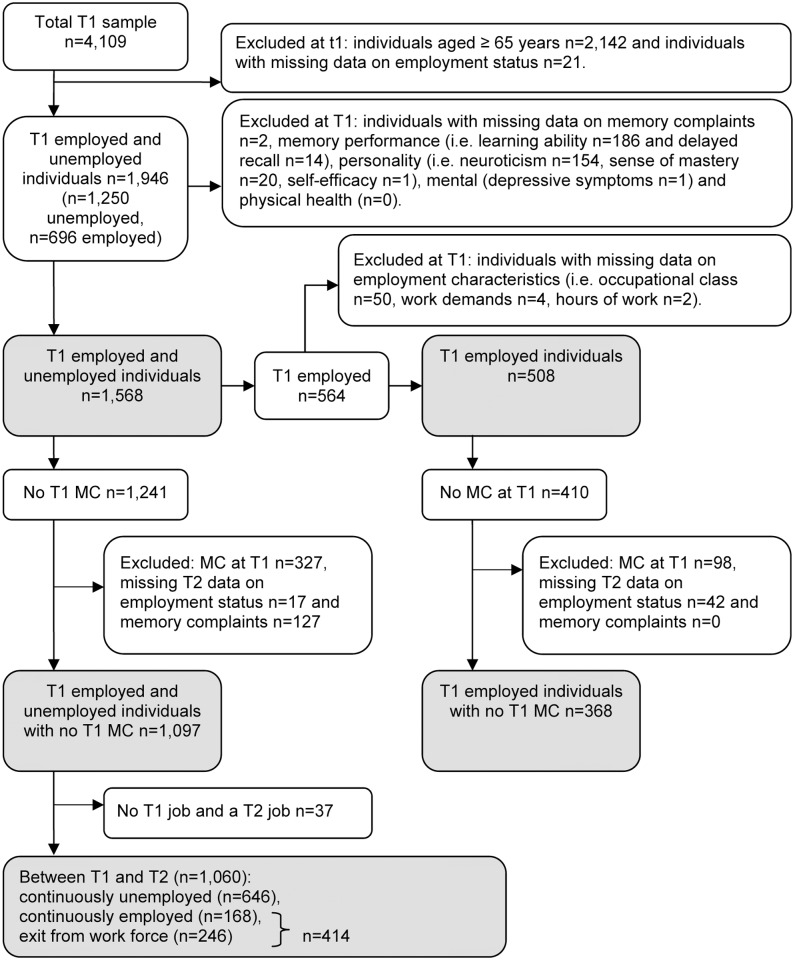
Flow-chart of selected respondents at baseline (T1) and three-year follow-up (T2).

Data from the first and second birth cohort were pooled (n = 4,109) for the current study. The 1992–93 and 2002–03 cycles were considered as baseline and the 1995–96 and 2005–06 cycles as follow-up. Respondents with missing data on employment status (n = 21) were excluded (see Fig. 1). Respondents aged ≥ 65 years (n = 2,142) were excluded because the mandatory retirement age in the Netherlands was 65 years at the time. A relatively large number of respondents are aged ≥ 65 years because the LASA study focuses on individuals aged 55–85 years and respondents were drawn from a population registry as opposed to for instance a job registry. Note that about 35.2% (n = 696) of n = 1,976 (4,109–2,142) respondents aged <65 years were employed, which correspondents to Dutch workforce participation data: available data from Statistics Netherlands show that the workforce participation of Dutch 55–64-year-olds was approximately 26% in 1996 and 37% in 2003 [15]. To examine the association between employment status and memory complaints, respondents with missing data at baseline (n = 378) were additionally excluded, resulting in an analytic sample of n = 1,568. To examine the longitudinal association between employment status at baseline and incident memory complaints at follow-up, respondents were additionally excluded who reported to have memory complaints at baseline (n = 327) and who had missing data at follow-up (n = 144). This resulted in an analytic sample of n = 1,097. No significant (p<0.05) differences were found between respondents who were lost to follow-up (i.e. respondents without follow-up data; n = 144) and respondents who remained in the study regarding gender, level of education, baseline age, employment status or memory complaints.

To examine the cross-sectional association between employment characteristics and memory complaints, respondents were selected who were employed (≥1 hours weekly; n = 564; Fig. 1). Respondents with missing data on employment characteristics (n = 56) at baseline were excluded. Thus, an analytic sample of n = 508 was used. The longitudinal association between employment characteristics and memory complaints was studied in respondents without memory complaints at baseline (n = 410) and respondents were excluded with missing data on employment status (n = 42). Therefore, an analytic sample of n = 368 was examined. No significant differences were found for respondents who were lost to follow-up (i.e. respondents without follow-up data; n = 42) compared to respondents with data at both waves regarding gender, level of education, baseline age, employment status or memory complaints.

### Dependent variable

The presence of memory complaints was assessed with the question ‘Do you have complaints about your memory?’ (Yes/No). This question has repeatedly been shown to be a pragmatic tool for identification of individuals with cognitive impairment, cognitive deterioration, and dementia (e.g. [3, 16, 17]). Respondents had prevalent memory complaints if they had memory complaints at baseline. Respondents had incident memory complaints if they had no memory complaints at baseline and memory complaints at follow-up.

### Independent variables

**Employment status**. Employment status was categorised into unemployed (0) and employed (1). Unemployed respondents included respondents who were looking for work, had never worked, received a disability pension, and were retired. Employed respondents had a paid job of at least one hour weekly. An additional employment status variable was computed to examine change of employment, which was categorised as 0) continuously employed (employed at both waves), 1) continuously unemployed (unemployed at both waves), and 2) left the workforce at follow-up (employed at baseline and unemployed at follow-up). Few respondents were unemployed at baseline and employed at follow-up and had memory complaints at follow-up (n = 7) and were therefore excluded. To examine employment trajectories in employees only, 0) continuously employed (employed at baseline and follow-up) were compared with 1) respondents who left the workforce at follow-up (employed at baseline and unemployed at follow-up).

### Employment characteristics

We explored four employment characteristics: high job level, job prestige, long working hours, high psychological demands, and iso-strain.

*Job level*—The occupation performed by a respondent was coded according to the Netherlands Standard Classification of Occupations 1992 (NSCO92) from Statistics Netherlands. Job level was derived from the NSCO92and ranged from 1 (elementary) through 5 (scientific) and was determined by Statistics Netherlands according to the level of required skills (i.e. elementary or scientific) which was based on the necessary level of education, trial period, and work experience.

*Job prestige*—Using the NSCO92, a job prestige scale was computed, developed by Sixma and Ultee [18] who determined this scale by asking Dutch individuals what level of prestige they think certain jobs have in society. Observed scores ranged from 13 (low; garbage collector) to 78 (high; surgeon).

*Weekly working hours*—Respondents were asked how many hours they worked per week (range 1 to 100). Hours of work was categorised into tertiles, i.e. 0) 1–21.2, 1) 21.3–40, and 2) 40.1 and higher because a non-linear association with memory complaints was found.

*Psychological job demands*—Examined job demands were task requirements (i.e. work fast, much work, work hard, hectic work) and cognitive demands (i.e. intensive thinking, need to keep focused, requiring much concentration). Occupational classes, derived from the NSCO92, were categorised to have low, moderate, and high probability of exposure to task requirements by applying a general population job-exposure matrix (GPJEM) [19]. Due to low numbers of employees with prevalent (n = 7) and incident (n = 2) memory complaints with high probability of exposure to task requirements, categories were collapsed into low (0) and moderate (1; moderate and high) probability in the current study. Occupational classes were only categorised to have either low (0) or high (1) probability of exposure to cognitive demands; no occupational classes exist that have moderate probability of exposure to cognitive demands.

*Iso-strain*—Work stress has been argued to not result from single work characteristics but from a joint effect of job demands and resources [20]. Resources are thought to counter any negative health effects of psychological job demands. Iso-strain jobs, i.e. a combination of high psychological demands and low psychosocial resources, have been considered to most likely result in stress [20, 21] compared to jobs with other combinations of demands and resources. Selected psychosocial resources were autonomy (e.g. decide how and when to perform a job), task variation (e.g. learn new things), co-worker support (e.g. my colleagues help to get the job done) and supervisory support (e.g. my supervisor helps to get the job done). Similar to the determination of job demands, jobs were categorised to have low (0) and moderate (1) probability of exposure to these resources by applying a GPJEM [19]. Jobs with both moderate probability of high demands and low probability of resources were regarded as iso-strain jobs in the current study.

### Covariates

In addition to demographic covariates, four groups of covariates potentially associated with the determinants and outcome were identified based on the literature: memory performance [3, 4, 22], physical health [6, 9, 23–27], mental health [4, 6, 24, 28–31], and personality traits [6, 32–37].

Age (years) and gender (men and women) were examined. Level of education was categorised into low (elementary school or less), medium (lower vocational, general intermediate, intermediate vocational, and general secondary education), and high (higher vocational education, college, and university).

A Dutch version of the Auditory Verbal Learning Test [38] was used to determine memory performance, i.e. learning ability and delayed recall. The test is limited to three (instead of five) trials to lighten the burden for respondents in LASA. During each trial, respondents are asked to recall as many words as possible of a total of 15 words. The total number of words learned by the respondent on all three trials, is defined as learning ability. After approximately 20 minutes, the respondent was asked to recall as many words as possible to measure delayed recall.

The presence of cardiovascular disease and multimorbidity was studied to measure physical health. If self-reported diseases or abnormalities to the arteries, heart disease or cerebrovascular accident were present, cardiovascular disease was coded as present (yes) as opposed to not present (no). Multimorbidity was examined by the number of chronic diseases and determined by the presence of seven self-reported major chronic diseases (chronic obstructive pulmonary disease, heart disease, peripheral arterial disease, stroke, diabetes mellitus, rheumatoid arthritis/osteoarthritis, cancer). These were selected based on their prevalence (>5%) in the 55+ age group in the Netherlands [39].

Mental health was measured by the presence of depressive symptoms as assessed by the Dutch translation of the Center for Epidemiologic Studies Depression Scale (CES-D) [40, 41]. The CES-D is a 20-item self-report scale, designed to measure depressive symptoms in the general population.

Personality traits sense of mastery, self-efficacy, and neuroticism were studied and were assessed by using a shortened version of the Pearlin and Schooler Mastery scale [42], the 12-item version of the Perceived Self-Efficacy Scale (PSES) [43, 44], and a shortened version (15 items) of the Dutch Personality Questionnaire [45], respectively. Higher scores indicated a higher level of sense of mastery, self-efficacy, and neuroticism.

### Statistical analysis

All analyses were weighed according to the Dutch population, for which demographic data according to sex and age from Statistics Netherlands were used. LASA data from the first birth cohort were weighed according to the Dutch population in 01–01–1993 and data from the second birth cohort were weighed according to 01–01–2003. Differences between sample characteristics of individuals with and without memory complaints were studied by applying the Chi-square test for categorical variables and the Independent samples T-test for continuous variables. Whether the prevalence and incidence of memory complaints differed between employed and unemployed individuals was examined by using the Chi-square test.

Logistic regression analysis was applied to assess the association of employment status with prevalent and incident memory complaints, and employment trajectories with incident memory complaints. Analyses were adjusted for relevant covariates. Covariates were considered relevant if an association (criterion: p<0.20) with the determinant and the outcome existed and, the regression coefficient of the determinant changed >10% after including the covariate in the model. The covariate that caused the largest percentage of change was subsequently added to the model and the remaining covariates were then tested again according to the stated criteria until all relevant covariates were included.

Two sensitivity analyses were performed. The association of baseline employment characteristics with incident memory complaints may be influenced by job status at follow-up. Therefore, it was examined whether leaving the workforce was an effect modifier. Also, it can be argued that jobs with high job level, high job prestige, long hours of work, high psychological demands, and high iso-strain are more likely to be jobs that are performed by individuals who attained a high level of education. Therefore, associations were additionally adjusted for level of education to determine whether our results may be explained by level of education.

## Results

### Sample characteristics

Sample characteristics are shown in Table 1. Middle-aged individuals with prevalent memory complaints were significantly older, had lower delayed recall, more depressive symptoms, and were more likely to have chronic diseases and a cardiovascular disease compared to those without prevalent memory complaints. Furthermore, they had lower self-efficacy, sense of mastery, and higher neuroticism scores compared to those without prevalent memory complaints. Compared to individuals without incident memory complaints, those with incident memory complaints had significantly lower learning ability, delayed recall, more depressive symptoms, were more likely to have chronic diseases, and had lower sense of mastery and higher neuroticism scores. Employed 55–64-year-olds with prevalent memory complaints and employed 55–64-year-olds with incident memory complaints worked significantly fewer hours weekly compared to those without prevalent and incident memory complaints respectively.

**Table 1:** Sample characteristics at baseline for those with and without memory complaints.

Sample characteristics at baseline		Prevalent memory complaints			Incident memory complaints		
Employed and unemployed 55–64-year-olds		No	Yes	p	No	Yes	p
Men, n (%)		609 (50.1)	139 (44.6)	.079	458 (50.6)	75 (45.5)	.228
Age (55–64), mean (SD)		59.6 (2.8)	60.0 (3.0)	.042	59.6 (2.8)	59.6 (2.8)	.891
Level of education	Low, n (%)	270 (22.2)	77 (24.6)	.638	200 (22.1)	34 (20.6)	.514
	Intermediate, n (%)	714 (58.8)	176 (56.2)		524 (57.8)	103 (62.4)	
	High, n (%)	231 (19.0)	60 (19.2)		182 (20.1)	28 (17.0)	
Employed (≥1 hours weekly), n (%)		477 (39.3)	110 (35.1)	.182	367 (40.5)	62 (37.6)	.486
Learning ability (0–39), mean (SD)		21.4 (5.6)	20.7 (5.6)	.068	21.8 (5.6)	20.5 (4.9)	.006
Delayed recall (0–15), mean (SD)		6.5 (2.7)	6.1 (2.7)	.040	6.7 (2.7)	5.9 (2.3)	.000
Depressive symptoms (0–48), median [IQR]		4.0 [2–9]	9.0 [4–15]	.000	4.0 [2–8]	6.0 [3–11]	.001
Number of chronic diseases	0, n (%)	633 (52.1)	108 (34.5)	.000	492 (54.3)	74 (44.8)	.010
	1, n (%)	417 (34.3)	132 (42.2)		305 (33.7)	58 (35.2)	
	≥2, n (%)	165 (13.6)	73 (23.3)		109 (12.0)	33 (20.0)	
Cardiovascular diseases, n (%)		185 (15.2)	75 (24.0)	.000	137 (15.1)	26 (15.8)	.834
Self-efficacy (21–60), mean (SD)		44.0 (5.3)	41.1 (5.9)	.000	44.2 (5.2)	43.5 (5.6)	.120
Sense of mastery (5–25), mean (SD)		18.5 (3.2)	16.8 (3.6)	.000	18.7 (3.1)	17.9 (3.5)	.006
Neuroticism (0–29), median [IQR]		4.0 [1–8]	8.0 [4–12]	.000	4.0 [1–7]	6.0 [2–9]	.000
Total, n (%)		1215 (100)	313 (100)		907 (100)	165 (100)	
**Employed 55–64-year-olds**							
Weekly hours of work	1–21.2, n (%)	131 (29.6)	44 (43.6)	.030	93 (28.7)	26 (46.4)	.015
	21.3–40.0, n (%)	226 (53.9)	40 (39.6)		179 (55.2)	20 (35.7)	
	40.1–100, n (%)	68 (16.0)	17 (16.8)		52 (6.0)	10 (17.9)	
Job level (1–5), mean (SD)		2.85 (1.00)	2.95 (1.03)	.408	2.9 (1.0)	2.7 (1.0)	.107
Job prestige (17–78), mean (SD)		39.9 (15.23)	40.3 (15.8)	.775	40.2(15.4)	38.2 (14.2)	.380
Moderate task requirements, n (%)		115 (27.1)	33 (32.0)	.285	94 (29.1)	10 (17.9)	.082
High cognitive demands, n (%)		113 (26.5)	35 (34.7)	.105	91 (28,1)	11 (20.0)	.211
Iso-strain, n (%)		98 (23.1)	24 (23.8)	.890	80 (24.7)	9 (16.1)	.160
Total, n (%)		425 (100)	101 (100)		324 (100)	56 (100)	

Note: IQR = Inter Quartile Range used for non-normally distributed scales. Prevalent MC: MC at baseline. Incident MC: no MC at baseline and MC at follow-up. Weighed according to the Dutch population. Therefore numbers may not always be in accordance with flow-chart.

In total, 20.5% reported memory complaints at baseline (Table 2). Among those without memory complaints at baseline, 15.4% had incident memory complaints. Prevalence and incidence of memory complaints did not differ significantly in unemployed compared to employed respondents.

**Table 2:** Weighed number of prevalent and incident MC in employed and unemployed individuals.

		Employment status at baseline		
		Unemployed	Employed	P^*1*^
**Prevalent MC, n (%)**	No, 1215 (79.5)	738 (78.4)	477 (81.3)	0.182
	Yes, 313 (20.5)	203 (21.6)	110 (18.7)	
	Total, 1528 (100)	941 (61.6)	587 (38.4)	
**Incident MC, n (%)**	No, 907 (84.6)	540 (84.0)	367 (85.5)	0.486
	Yes, 165 (15.4)	103 (16.0)	62 (14.5)	
	Total, 1072 (100)	643 (60.0)	429 (40.0)	

^1^Chi-square test

Note: MC = memory complaints. Prevalent MC: MC at baseline. Incident MC: no MC at baseline and MC at follow-up. Weighed according to the Dutch population. Therefore numbers may not always be in accordance with flow-chart.

### The association of employment status and employment characteristics with prevalent and incident memory complaints

No significant association was found between baseline employment status and baseline prevalent memory complaints (Table 3). In addition, employment status at baseline and employment trajectories were not significantly associated with incident memory complaints after three years.

**Table 3:** Associations of employment with prevalent MC at baseline and incident MC at (3-year) follow-up.

			Prevalent MC (unadjusted models)				Prevalent MC (adjusted models^1^)			
Employment status and trajectories		Covariates	OR	CI	p	R^2^	OR	CI	p	R^2^
**Employed**	**≥1 hours weekly vs. unemployed**		0.84	0.65–1.09	.181	.002	1.08	0.82–1.42	.582	.092
		Neuroticism (0–29)	.	.	.		1.11	1.09–1.13	.000	
			**Incident MC (unadjusted models)**							
**Employed**	**≥1 hours weekly vs. unemployed**		0.89	0.63–1.25	.507	.001	.	.	.	.
**Employment trajectories**	**Continuously unemployed vs. continuously employed**		1.08	0.72–1.62	.710	.001	.	.	.	.
	**Left the work force at follow-up vs. continuously employed**		0.93	0.53–1.62	.806		.	.	.	

Note: R^2^ = Nagelkerke R square. MC = memory complaints. Prevalent MC: MC at baseline. Incident MC: no MC at baseline and MC at follow-up. Weighed according to the Dutch population.

^1^ Models are shown only if relevant covariates were found.

The association between hours worked weekly and prevalent memory complaints was lost after including depression, which was the only relevant covariate (Table 4). Furthermore, multivariate analyses adjusted for self-efficacy, cardiovascular disease, and delayed recall showed that individuals who performed jobs with high probability of exposure to cognitive demands were more likely to have memory complaints compared to individuals who performed a job with low probability of exposure to cognitive demands (OR = 1.98, CI = 1.20–3.26, p = .007; note that the effect size was small, i.e. R^2^ = .125). No other significant associations were found between employment characteristics and prevalent memory complaints, regardless of inclusion of relevant explanatory variables.

**Table 4:** Associations of baseline employment characteristics with prevalent MC at baseline and incident MC at (3-year) follow-up.

Employment characteristics		Covariates	Prevalent MC (unadjusted models)				Prevalent MC (adjusted models^1^)			
			OR	CI	p	R^2^	OR	CI	p	R^2^
**Job level (1–5)**		.	1.10	0.88–1.36	.407	.002	.	.	.	.
**Job prestige (0–78)**		.	1.00	0.99–1.02	.774	.000	.	.	.	.
**Weekly hours of work**	**21.3–40.0 vs. 1–21.2**		0.53	0.33–0.85	.008	.021	0.63	0.38–1.05	.076	.133
	**40.1–100 vs. 1–21.2**		0.73	0.39–1.37	.325		0.99	0.50–1.92	.964	
		Depressive symptoms (0–48)	.	.	.		1.12	1.08–1.17	.000	
**Task requirements**	**Moderate vs. low**		1.27	0.80–2.03	.314	.003	.	.	.	.
**Cognitive demands**	**High vs. low**		1.44	0.91–2.29	.122	.007	1.98	1.20–3.26	.007	.125
		Self-efficacy (21–60)	.	.	.		0.89	0.85–0.94	.894	
		Cardiovascular diseases	.	.	.		2.07	0.95–4.52	.067	
		1 chronic diseases vs. 0	.	.	.		1.81	1.08–3.05	.025	
		≥2 chronic diseases vs. 0					1.43	0.60–3.41	.421	
**Iso-strain**	**Yes vs. no**		1.05	0.63–1.74	.863	.000	.	.	.	.
			**Incident MC (unadjusted models)**				**Incident MC (adjusted models** ^**1**^)			
**Job level (1–5)**			0.79	0.59–1.05	.108	.012	0.82	0.61–1.11	.205	.049
		Delayed recall (0–15)	.	.	.		0.85	0.75–0.96	.006	
**Job prestige (0–78)**			0.99	0.97–1.01	.379	.004	.	.	.	
**Weekly hours of work**	**21.3–40.0 vs. 1–21.2**		0.39	0.21–0.74	.004	.039	0.35	0.18–0.67	.002	.087
	**40.1–100 vs. 1–21.2**		0.66	0.29–1.48	.309		0.65	0.29–1.49	.310	
		Delayed recall (0–15)	.	.	.		0.83	0.73–0.93	.002	
**Task requirements**	**Moderate vs. low**		0.53	0.26–1.10	.088	.015	0.64	0.30–1.33	.230	.049
		Delayed recall (0–15)	.	.	.	.	0.85	0.76–0.96	.009	
**Cognitive demands**	**High vs. low**		0.66	0.33–1.32	.243	.007	.	.	.	
**Iso-strain**	**Yes vs. no**		0.57	0.26–1.22	.145	.011	0.66	0.30–1.44	.297	.047
		Delayed recall (0–15)	.	.	.		0.85	0.75–0.96	.007	

^1^ Models are shown only if relevant covariates were found.

Note: R^2^ = Nagelkerke R square. MC = memory complaints. Prevalent MC: MC at baseline. Incident MC: no MC at baseline and MC at follow-up. Weighed according to the Dutch population.

Furthermore, a significant association was found between hours worked weekly and incident memory complaints after adjustment for delayed recall; individuals who worked 21.3–40 hours weekly were less likely to develop memory complaints compared to individuals who worked less than 21.3 hours weekly (OR = 0.35, CI = 0.18–0.67, p = .002; note that the effect size is very small R^2^ = .087; Table 4). No other significant independent associations with incident memory complaints were found.

Sensitivity analyses showed that leaving the workforce was not a significant effect modifier in any of the associations between employment characteristics and incident memory complaints. In addition, level of education did not explain the examined associations of employment status and employment characteristics with prevalent and incident memory complaints.

## Discussion

Whether employment status was associated with prevalent memory complaints and predicted incident memory complaints after three years was first examined. No such association was found, which corroborates the results from Derouesné and colleagues [10].

Second, we examined whether specific employment characteristics were associated with memory complaints. The results showed that individuals employed in work with high probability of cognitive demands were more likely to report memory complaints. Jobs with high cognitive demands, task requirements or iso-strain are all thought to be important sources of stress [20]. Although we can only speculate, work-stress alone does not seem to explain why cognitive demands are associated with memory complaints. Task requirements and iso-strain were not found to be associated with memory complaints, so an additional explanation is needed. As argued by Aarts and colleagues [9], employed older individuals may more often have memory complaints compared to older individuals who are not employed, because their work requires good memory performance and they are therefore confronted with their memory performance. This might particularly be true for older individuals with cognitively demanding work. As opposed to task requirements (i.e. work fast, much work, work hard, hectic work) and iso-strain, particularly cognitive demands, such as intensive thinking, need to keep focused, and requiring much concentration, may be demanding on (not necessarily failing) memory performance. The demands on their memory in their work consequently directs their attention towards their memory performance. As a result, individuals with cognitively demanding jobs may simply be more likely to notice their deteriorating but not necessarily failing memory performance than individuals who do not work in cognitively demanding jobs. Note that objective memory performance did not prove to affect the relationship between cognitive demands and memory complaints. Additional sensitivity analyses (results not tabulated) showed that having a cognitively demanding job compared to not having a cognitively demanding job was significantly associated with higher delayed recall performance (Pearson r = 0.186, p = .000) and learning ability (Pearson r = .206, p = .000). This underlines that their memory complaints are unlikely to represent objectively measured poor memory performance.

If older workers with cognitively demanding jobs report prevalent memory complaints because they simply notice their deteriorating memory performance due to their cognitive work demands, these individuals may benefit from a decrease in cognitive work demands to reduce memory complaints. In addition, memory complaints have been shown to also be related to executive functioning in employed individuals [46]. Therefore, individuals performing cognitively demanding jobs may also benefit from lowering distractions from the working environment. Still, more research is needed to determine whether a relationship with executive functioning exists in particularly older workers.

No association was found between incident memory complaints and cognitive demands. On the one hand, this may suggest that this employment characteristic affects memory complaints acutely, rather than affect memory complaints after a longer period of time [13]. On the other hand, it is unknown whether prevalent memory complaints were persistent, i.e. already present for a longer period time. If they were, it implies that these memory complaints developed at a younger age and, therefore, are not necessarily related to effects of cognitive ageing, but simply to strain put on memory performance. Still, comparisons between the samples used for prevalent and incident memory complaints should be interpreted with caution due to disparate sample sizes. To determine whether those memory complaints are or are not reported due to (subtle) effects of cognitive aging, research is needed examining whether memory complaints are reported by individuals performing jobs with cognitive demands at all ages.

Another finding was that those working fewer hours were more likely to report incident memory complaints. Possibly, this result points to a healthy-worker effect; individuals with poor memory performance, poor mental or physical health or memory complaints are more likely to work fewer hours. Although adjustments were made for relevant covariates, individuals working few hours may have had poor memory performance or (mental) health which was not yet measurable at baseline, but as a consequence started to work less.

Some limitations of the present study should be mentioned. First, it is unknown whether the examined memory complaints were persistent or transient, which may have biased our results. Second, it is unknown how often respondents were confronted with memory problems. This could have provided more information on the severity of the memory complaints and may be explored in future studies. Third, we examined whether the relationship between employment characteristics and memory complaints differed for those who left the workforce and those who did not. However, it was not examined whether exposure to employment characteristics had changed during follow-up, for instance because of job change. This may explain why no relationship was found between employment characteristics and memory complaints. Still, demotion (i.e. a decline in function type and salary) is very uncommon in the Netherlands [47] and therefore unlikely to have biased our results. Fourth, the job prestige scale applied in the current study was developed in the 1980s. The assigned prestige levels may no longer be up-to-date and research is necessary to better capture today’s job prestige. Fifth, we classified individuals working 1 hour weekly as employed. Possibly, individuals working few hours weekly are not sufficiently exposed to employment (characteristics) for it to result in memory complaints. Therefore, we performed additional sensitivity analyses in which all examined associations were adjusted for hours of work. The results showed that hours of work did not affect our results, i.e. similar results for the relationship between prevalent memory complaints and job level (OR = 1.16, p = 0.197, CI = 0.93–1.44), job prestige (OR = 1.01, p = 0.431, CI = 0.99–1.02), task requirements (OR = 1.31, p = 0.261, CI = 0.82–2.11), cognitive demands (OR = 1.95, p = 0.009, CI = 1.18–3.22), and iso-strain (OR = 1.03, p = 0.903, CI = 0.62–1.73). Also similar results were found for the relationship between incident memory complaints and job level (OR = 0.91, p = 0.533, CI = 0.67–1.24), job prestige (OR = 1.00, p = 0.788, CI = 0.98–1.02), task requirements (OR = 0.70, p = 0.346, CI = 0.33–1.48), cognitive demands (OR = 0.66, p = 0.238, CI = 0.33–1.32), and iso-strain (OR = 0.65, p = 0.288, CI = 0.30–1.44). As individuals who were not employed did not have data on hours of work, adjusting for hours of work was not possible. In this case, we examined the associations without the respondents that worked 1–21.2 hours weekly. Excluding these individuals did not affect the relationship of prevalent memory complaints with employment status (OR = 0.89, p = 0.471, CI = 0.64–1.23) and the relationship of incident memory complaints with employment status (OR = 1.47, p = 0.068, CI = 0.97–2.23) and employment trajectories (continuously unemployed vs. continuously employed: OR = 1.37, p = 0.227, CI = 0.82–2.30; left the workforce at follow-up vs. continuously employed: OR = 0.77, p = 0.518, CI = 0.35–1.69). Sixth, we measured work demands by using a general population job-exposure matrix (GPJEM). A disadvantage of GPJEMs in general is their inability to account for exposure heterogeneity within the job categories. Using self-reported work exposures may provide more precise information on individual work exposures. However, self-reported work exposures may be influenced by individual factors (e.g. mood, past experiences, health) [48]. An advantage of aggregating self-reported work exposures, as done for the development of the JEM applied, is that the influence of such individual factors may be reduced. More research is needed to determine whether the relationship between work demands and memory complaints is affected by the use of a different method of measuring work demands. Finally, the association between cognitive demands and prevalent memory complaints was adjusted for self-efficacy, CVD, and number of chronic diseases. As both CVD and number of chronic diseases were included in this model, it was examined whether a risk of collinearity existed as a sensitivity analysis (results not tabulated). No evidence was found for collinearity: excluding either CVD or number of chronic diseases from the analysis resulted in similar results for cognitive demands (OR = 1.87, p = 0.012 after excluding CVD and OR = 1.90, p = 0.011 after excluding number of chronic diseases).

An important strength of the current study is that this is the first study to examine the association between employment (characteristics) and specifically memory complaints in a representative sample of middle-aged workers and individuals still eligible for the labour force. We were additionally able to explore various employment characteristics, while adjusting for a wide range of covariates known to underlie or cause memory complaints, including objectively measured memory performance.

Prevalence of memory complaints has scarcely been examined in 55–64-year-olds [1, 49]. No studies that we are aware of examined incident memory complaints. Results from a previous Dutch study revealed a higher prevalence of reported memory complaints in 55–64 year-olds, namely 41% [1]. They used a different question to examine memory complaints (‘Do you consider yourself as being forgetful?’) compared to our question which may explain the disparate prevalence rates. We namely found that one in five people (i.e. 20.5%) reported to have memory complaints. Another 15.4% developed memory complaints after three years. For older individuals employed or still eligible for the workforce, the prevalence and incidence of memory complaints seem quite substantial. Those prevalence and incidence numbers were not adjusted for any underlying or causal factors, so memory complaints may be an indication of poor cognitive performance, physical or mental health problems, which in turn may inhibit individuals to continue employment at higher ages.

Our most important finding was that cognitive demands may result in memory complaints, when underlying or causal factors of memory complaints, such as memory performance and health problems, were accounted for. For these individuals, it is important to decrease memory complaints because memory complaints have been shown to cause some obstruction in daily functioning [1] and lower life satisfaction between age 55 to 91 and even more so in those aged 55 to 69 years [2]. Also, as Metternich and colleagues [50] argued, memory complaints may disrupt daily occupational activity, which can become a secondary stress factor. Possibly, employers and employees have unreasonable expectations of 55–64-years-olds’ memory performance and jobs need to be adjusted to their cognitive capacity. Especially since this age group will more often be required to work until higher ages and jobs are more often demanding for cognitive performance, research is needed examining whether for instance cognitive training or even decreasing demands in late middle-aged individuals may decrease memory complaints.

## Supporting Information

S1 Dataset Dataset MC and Work. (SAV)

S1 Syntax Syntax Sample MC and Work. (SPS)
